# Changes in Metabolic Regulation and the Microbiota Composition after Supplementation with Different Fatty Acids in db/db Mice

**DOI:** 10.1155/2022/3336941

**Published:** 2022-01-07

**Authors:** Beatriz Elina Martínez-Carrillo, Talia Mondragón-Velásquez, Ninfa Ramírez-Durán, José Félix Aguirre-Garrido, Roxana Valdés-Ramos, Ana Laura Guadarrama-López, Arturo Castillo-Cardiel

**Affiliations:** ^1^. Laboratorio de Investigación en Nutrición, Facultad de Medicina, Universidad Autónoma del Estado de México, Paseo Tollocan, Esquina Jesús Carranza, s/n, Colonia Moderna de la Cruz, C.P, 50180 Toluca, Mexico; ^2^. Laboratorio de Microbiología Médica y Ambiental, Facultad de Medicina, Universidad Autónoma del Estado de México, Paseo Tollocan, Esquina Jesús Carranza, s/n, Colonia Moderna de la Cruz, C.P, 50180 Toluca, Mexico; ^3^Laboratorio de Biotecnología Ambiental, Departamento de Ciencias Ambientales, Universidad Autónoma Metropolitana, Unidad Lerma, Lerma de Villada, Estado de México, Mexico; ^4^Departamento de Cirugía Experimental, Universidad Quetzalcoátl de Irapuato, Blvd. Arandas No. 975 Colonia Tabachines, C.P. 36715, Irapuato, Guanajuato, Mexico

## Abstract

**Introduction:**

The effects of fatty acids on health vary and depend on the type, amount, and route of consumption. EPA and DHA have a defined role in health, unlike coconut oil.

**Objective:**

The aim was to investigate the changes in metabolic regulation and the composition of the culture-dependent microbiota after supplementation with different fatty acids in db/db mice. *Material and Methods*. We were using 32 8-week-old db/db mice, supplemented for eight weeks with EPA/DHA derived from microalgae as well as coconut oil. The lipid, hormonal profiles, and composition of the culture-dependent microbiota and the phylogenetic analysis based on the 16S rRNA gene sequencing were determined for identification of the intestinal microbiota.

**Results:**

Enriched diet with EPA/DHA reduced TNF-*α*, C-peptide, insulin resistance, resistin, and the plasma atherogenic index, but increased TC, LDL-c, VLDL-c, and TG without changes in HDL-c. Coconut oil raised the HDL-c, GIP, and TNF-*α*, with TG, insulin resistance, adiponectin, and C-peptide reduced.

**Conclusion:**

The most abundant microbial populations were *Firmicutes* and the least *Proteobacteria*. EPA/DHA derived from microalgae contributes to improving the systemic inflammatory status, but depressed the diversity of the small intestine microbiota. Coconut oil only decreased the C-peptide, raising TNF-*α*, with an unfavorable hormonal and lipid profile.

## 1. Introduction

Type 2 diabetes mellitus (T2DM) is a metabolic disorder in which glucose metabolism cannot be regulated [1]. This condition is associated with a proinflammatory state that involves overproduction of cytokines such as IL-6, IL-1, and tumor necrosis factor-*α* (TNF-*α*). This hinders the interaction of insulin with its receptor causing resistance to insulin and organic complications [2]. A sedentary lifestyle and excessive energy consumption increase body weight [3] to exacerbate the inflammatory metabolic state that generates metabolic syndrome [4]. The role of the type of gut microbiota on the inflammatory state of T2DM is controversial [5, 6]. The intestinal microbiota is a dynamic entity that participates in the maturation of the local immune system, drug metabolism, detoxification, and vitamin production. The microbiota can prevent the adhesion of pathogenic bacteria [7–9]. It is also associated with metabolic disorders because it increases the energy harvesting of the diet, modifies the host's genetic expression, and increases metabolic endotoxemia and inflammation [10]. The gut microbiota is susceptible to modifications by various factors such as lifestyle, type of diet consumed, antibiotics, and genetic factors [6, 11, 12]. All of these factors affect the prevalence, abundance, or scarcity of bacterial species [13, 14]; therefore, the interaction of local species with the content of the small intestine and the modification of these factors has become very important, since together they can improve the metabolic and inflammatory status, as well as the quality life of patients with T2DM [15–17].

The microbiota increases in abundance and diversity from the gastric lumen to the small intestine and to the colon/rectum where it reaches its maximum concentration [18]. The resident microbiota in the colon are the most abundant and have been widely studied due to the easy collection [14]; little is known regarding the composition of the microbiota in the small intestine [19, 20].

The quality and quantity of fat in the diet can modify features that impact health such as the composition of the intestinal microbiota and some metabolic and immunological pathways. Dietary fats can be classified according to their degree of saturation: saturated fatty acid (SFA), monounsaturated fatty acid (MUFA), and polyunsaturated fatty acids (PUFA). Coconut oil contains about 90% of saturated fatty acids [21]. Its use is still controversial; while it increases cardiovascular risk, there is also evidence that it reduces body weight and glycaemia [22]; little is known about its antidiabetic potential and its influence on the gut microbiota [17]. In contrast, *n-3* PUFAs have a more defined role in the modification of these parameters [7], like anti-inflammatory, antiarrhythmic, immunoprotective, cytoprotective, neuroprotective, and antiapoptotic functions [23]. The family of *n-3* PUFAs includes eicosapentaenoic acid (EPA, 20 : 5 *n-3*) and docosahexaenoic acid (DHA, 22 : 6 *n-3*), which are derived from alpha-linolenic acid (ALA, 18 : 3 *n-3*) [24]. Both are incorporated into the phospholipids of the cell membrane [25, 26]. They are cytoprotectors with anti-inflammatory activity, and they may be synthesized from ALA but in very small quantities; therefore, they must be consumed as part of the diet [27]. Thus, EPA and DHA are considered semiessential in relation to other nutrients [28]. They are mainly derived from marine animals and plants, and their most common source is oils from fish (25 to 30%) [27] or marine algae (8%) [29]. Microalgae are currently considered a rich and renewable source of EPA and DHA [30]; they are organoleptically more acceptable and a better alternative [31, 32] than fish oil [33]. In general, *n-*3 PUFAs have shown positive effects on the resident microbiota [34] because they suppress the production of inflammatory cytokines, which in turn modify the intestinal microbiota to reduce metabolic endotoxemia [35]. This leads to an increase in beneficial microorganisms for protection against gastrointestinal diseases [36, 37]. Most existing studies focus on the composition and function of the microbiota from the large intestine as well as on the effects of EPA and DHA derived from fish oil; therefore, this study explored the changes in metabolic regulation and the composition of the culture-dependent microbiota after supplementation with different fatty acids in db/db mice.

## 2. Materials and Methods

### 2.1. Study Design

This is an experimental intervention, cross-sectional, and controlled study. Thirty-two 8-week-old male mice of the db/db strain were used. The db/db strain (BKS.Cg+Leprdb+LeprdbOlaHsd Harlan®, Indianapolis, Indiana, USA) was obtained from Harlan® Laboratories. The study was carried out in accordance with the ARRIVE guidelines and the Mexican Ministry of Agriculture procedures (NOM-062-ZOO-1999) [38]; the protocol has been approved by the Research Ethics Committee of the UAEMex (approval number 008/2015). Mice were housed in groups of 4 *per* cage throughout the experiment under controlled conditions with a temperature of 19 to 21°C and light/dark cycles of 12/12 h. The experiments were performed with 8-week-old mice for 8 weeks (until week 16). Food and water were administered *ad libitum* throughout the experiments. Food consumption was verified by weighing the food placed in the cage and subtracting the food consumed daily to calculate the consumption *per* mouse.

### 2.2. Experimental Groups

**Figure 1:**
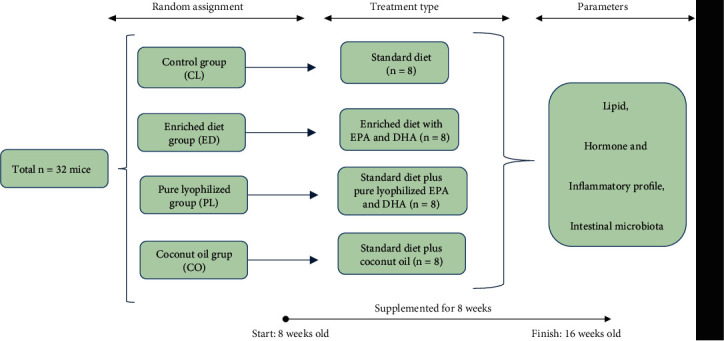
Distribution of study groups, based on strain type, form, and timeline of supplementation administered. Study groups: control group (CLG), enriched diet group (EDG), lyophilized group (LG), and coconut oil group (COG).

The 32 mice were distributed in four groups (*n* = 8) and divided according to the type of fatty acids administered: (i) control group (CLG) was without treatment and fed a standard diet, (ii) enriched diet group (EDG) has a modified meal that contains the same nutritional balance as the standard upon addition of 2.0% EPA/DHA derived from microalgae (Table 1), (iii) lyophilized group (LG) uses EPA/DHA derived from microalgae in the form of pure powder lyophilized with water as a vehicle plus the standard diet, and (iv) coconut oil group (COG) was administered pure coconut oil plus the standard diet (Figure 1). The standard diet was Rodent Laboratory Chow® 5001 from Purina (RLChow-5001, St. Louis, MO, USA) as shown in Table 1.

### 2.3. Source of *n-3* Fatty Acids (EPA/DHA)

The native microalgae were isolated and collected by BIOMEX, S.A. de C.V. (Guadalajara, Jalisco, Mexico) and belong to the microalgae families of *Chlorophyceae* and *Eustigmatophyceae*, which have a high content of EPA and DHA. BIOMEX carried out the culture, the biomass separation, and finally the chromatographic procedures to determine and obtain the lyophilized EPA and DHA (EPA [24%] + DHA [1.7%]) in the form of free fatty acids.

### 2.4. Supplementation

To compare whether the effect of administering the *n-3* PUFAs derived from microalgae mixed with the usual diet versus direct oral deposition administration causes modifications in metabolic regulation and the intestinal microbiota, three different forms of consumption were administered: two prepared from the lyophilized microalgae and one with coconut oil, as described below.

1. A pellet enriched with EPA/DHA was prepared with 2.0% more *n-3* fatty acids, 10 times more than the content of the normal diet (Table 1). The enriched food was placed in the cages for *ad libitum* consumption 24 hours a day
2. The lyophilized was reconstituted with ultrapure water and was administered daily at a dose of 1 mg/g of mouse weight by direct oral deposition with micropipette
3. Pure coconut oil (C1758-500 Sigma-Aldrich®, St. Louis, Missouri, USA) was administered daily at a dose of 1 mg/g of mouse weight by direct oral deposition with micropipette

### 2.5. Determination of Weight and Body Mass Index (BMI)

The body weight and nose to anus length were quantified weekly. The weight of the mice was obtained with a scale (Ohaus® Triple Beam 700/800 series). The nose to anus length was measured with a fiberglass tape. From these parameters, the body mass index (BMI) was calculated via the following formula: [weight (g)/length (cm)^2^∗100] [39].

### 2.6. Glucose Determination

Peripheral blood glucose concentration was determined weekly from the 8th to 16th week of the rodent's life. The sample was taken by puncture of the distal tail vein via capillary action using a Bayer One Touch® glucometer at 7:30 am before administering supplementation.

### 2.7. Plasma Collection Procedures

At the end of 16 weeks, the mice were sacrificed with sodium pentobarbital at a dose of 80 mg/kg. Blood was drawn by cardiac puncture with a syringe impregnated with 50 *μ*L of heparin. The blood was then centrifuged at 1500 rpm for 5 minutes, and the plasma was collected and stored in 1.5 mL tubes to quantify lipids, hormones, and inflammatory markers.

### 2.8. Metabolic and Inflammatory Profile

As part of hormonal and inflammatory profile, insulin, resistin, adiponectin, leptin, gastric inhibitory polypeptide (GIP), C-peptide, and TNF-*α* were quantified. For this, the commercial kits of Merck, Mouse Adipokine Standard (Cat. No. LMA-8071, Merck KGaA, Darmstadt, Germany) and Mouse Metabolic Hormone Detection Antibodies (Cat. No. MMH-1044, Merck KGaA, Darmstadt, Germany), were used.

### 2.9. Lipid Profile

Lipid profile was quantified by a colorimetric enzymatic method with reagents from the commercial house of Randox (Laboratories Ltd, County Antrim, UK), according to the supplier's specifications. A SELECTRA II device was used for the analysis and reading of the data. The quantified parameters were as follows: total cholesterol (TC, Cat. No. CHSL-0707), very low-density lipoprotein cholesterol (VLDL-c, Cat. No. VLDL-03322), low-density lipoprotein cholesterol (LDL-c: Cat. No. LDLL-0230), and high-density lipoprotein cholesterol (HDL-c, Cat. No. HDLL-0230). The plasma atherogenic index PAI = Log [TG/HDL‐c], cardiac risk ratio CRR = [CT/HDL‐c], and cardioprotective index CPI = [HDL‐c/LDL‐c] were obtained [40].

### 2.10. Obtaining Samples of Intestinal Content

The entire small intestine was removed from each mouse and placed in 1 mL of 1x phosphate buffer solution (1x PBS); it was then washed with 3 mL of 1x PBS to remove all of intestinal contents. The resulting liquid was centrifuged at 1500 rpm for 10 minutes. The supernatant was removed, and the solids from the contents of the small intestine were stored with 1 mL of physiological solution at -80°C until processing.

### 2.11. Obtaining Intestinal Microbiota

#### 2.11.1. Identification of Predominant Aerobic Bacteria in the Cultivable Microbiota

The intestinal content was diluted in 1 mL of physiological solution 0.9% (9 g of sodium chloride per liter of solution). Petri dishes were inoculated with 200 *μ*L of liquid fecal matter using the plaque extension method in brain heart infusion (BHI) agar medium (BD Bioxon Cat. No. 255003, Becton Dickinson, New Jersey, USA). The plates were incubated at 37°C for 24 to 48 hours under aerobic conditions. The predominant colonies were isolated and purified after the incubation time had elapsed.

#### 2.11.2. DNA Extraction Method

The strains were inoculated in brain heart infusion agar (BHI) by cross streak and were incubated at 37^o^ C for 18-24 hours. The biomass was recovered by scraping off the bacterial growth that had collected into micro tubes under sterile conditions. The commercial Promega Wizard® Genomic Kit (Cat. No. A1120, Massachusetts, USA) was used to obtain the genomic DNA following the manufacturer's instructions.

#### 2.11.3. Amplification of the 16S rRNA Gene

The Taq polymerase enzyme (MyTaq DNA Polymerase, Bioline, Cat. No. BIO-21105, Buenos Aires, Argentina) was used with the universal primers (8F: AGAGTTTGATCMTGGCTCAG and 1492R: TACGGYTACCTTGTTACGACTT) for polymerase chain reaction (PCR) and amplification of the 16SrRNA gene. A 1% agarose gel electrophoresis (Conda Pronadisa, Cat. No. 8100.10, Madrid, Spain) was used with ethidium bromide staining (BrEt, Sigma-Aldrich Cat. No. E7637-1G, St. Louis, Missouri, USA) to observe the fragments obtained from the amplification of the 16S rRNA gene (120 V for 40 min in TAE 1x Invitrogen buffer; Cat. No. 24710-030, California, USA).

#### 2.11.4. 16S rRNA PCR-RFLP Ribotyping

Digestion of 10 *μ*L of each amplicon of the 16S rRNA gene was carried out with the RSAI enzyme (Rhodopseudomonas sphaeroides Rsa-I, Promega, Cat. No. R6371, Massachusetts USA). To obtain the ribotyping, 7.6 *μ*L of nuclease-free water (Merck Millipore, Cat. No. LSKNF0500, Merck KGaA, Darmstadt, Germany), 0.2 *μ*L of B10X buffer (Promega, Cat. No. R002A, Massachusetts, USA), 0.2 *μ*L of acetylated bovine serum albumin (Promega, Cat. No. R396D, Massachusetts, USA), and 0.2 *μ*L of RSAI restriction enzyme were used. The final volume of the reaction was 20 *μ*L.

The MSPI enzyme (Moraxella sp. Msp-I, Promega, Cat. No. R6401, Massachusetts, USA) was used to digest 10 *μ*L of the amplicons of the 16S rRNA gene. In both cases, restriction was performed for 60 minutes at 37° C and was inactivated by heating for 15 minutes at 72° C. Restriction products were observed by 1.5% agarose gel electrophoresis with TAE 1x buffer (Invitrogen, Cat. 24710-030, California, USA) at 120 V for 80 min and stained with ethidium bromide. We were using a marker of 1 kb molecular weight of DNA (Thermo Scientific, Cat. No. 5M0311, Massachusetts, USA).

The resulting restriction patterns were analyzed according to the number of bands and the size with respect to the molecular weight marker used. Profiles with at least two bands were included for cluster analysis. The group of strains with identical enzyme restriction profiles was defined as a ribotype with the PyElph program version 1.4 [41]. The grouping was performed using the Sorensen-Dice coefficient and the unweighted pair group arithmetic method (UPGMA) to obtain a dendrogram as a graphic representation of the distance between the restriction patterns.

#### 2.11.5. Sequencing and Identification of Genera and Species

Here, 20% of the strains included in each ribotype were chosen to be genetically identified. A new amplification of the 16S rRNA gene of each strain was performed. Amplicons were purified with the Amicon Ultrafilter® Kit (Millipore, Cat. No. UFC500308, Merck KGaA, Darmstadt, Germany) and sent to the sequencing service in Macrogen, Maryland, USA. For species identification and phylogenetic analysis, the sequences obtained were analyzed with the following programs: BioEdit v7.2.5.0 [42], ClustalX2 v2.1 [43], Sea View v4.6.4 [44], and Mega7 v7. 0.26 [45]. Consensus sequences were constructed and compared with the sequences deposited in the GenBank of the National Center for Biotechnology Information (NCBI) through the BLAST program (basic tool for local alignment search) (https://blast.ncbi.nlm.nih.gov/) [46]. The presence of chimeric sequences was ruled out by the Bellerophon program [47]. For construction of the phylogenetic tree, the contiguous binding method was used with the aligned sequences and an initial analysis of 1000 repetitions [48]. The sequences deposited in the NCBI GenBank are listed in Table 2 of Results.

### 2.12. Statistical Analysis

A variance homogeneity test was applied, and measures of central tendency and dispersion were performed. Differences between mouse groups (enriched diet, lyophilized, and coconut oil) were analyzed. For homogeneous data, a comparison between groups used a one-way ANOVA with a Bonferroni *post hoc* test to determine intragroup differences. The lipid and hormonal profile data were not homogeneous; therefore, the Kruskal-Wallis nonparametric test was applied. Differences were considered significant with a *P* value < 0.05. The data were analyzed with SPSS v.23 software for Windows.

## 3. Results

### 3.1. Body Mass Index, Glycaemia, and Food Consumption

There were no significant differences for BMI between the supplemented groups and the control group (Table 3). In glycaemia, statistically significant differences were found between the enriched diet and coconut oil groups versus the control group (*P* < 0.001). There were no significant changes in the lyophilized group (*P* = 0.763). Finally, food consumption was lower in the enriched diet group and very high in the coconut oil groups with no changes in the lyophilized group, compared to the control group (*P* < 0.012).

### 3.2. Lipid Profile

Differences were found in the lipid profile between supplemented groups versus control group. The total cholesterol, LDL-c, VLDL-c, and TG were increased in the EPA- and DHA-enriched diet group with no changes in HDL-c, with the PAI decreased and the CPI increased with no changes in the CRR (Table 4). Supplementation with the lyophilized increased HDL-c in addition to TC, VLDL-c, and TG with no changes in LDL-c. This increased the behavior of PAI and CRR with a decrease in CPI (Table 4). Rather, the coconut oil administration increased TC, HDL-c, and LDL-c with a decrease in VLDL-c and TG, as well as atherogenic and cardioprotective index; this increased the CRR (Table 4).

### 3.3. Hormonal and Inflammatory Profile

Changes in hormonal and inflammatory profiles were observed in all groups (Table 5). The enriched diet group had a decrease in the secretion of insulin, resistin, adiponectin, C-peptide, and TNF-*α*, with an increase in GIP. This behavior was similar in the lyophilized group with a decrease in insulin, resistin, adiponectin, and C-peptide concentrations and significant increases in GIP and TNF-*α*. The comparison between the coconut oil group and the lyophilized group was similar with a decrease in the secretion of insulin, adiponectin, resistin, and C-peptide; the GIP and TNF-*α* increased, but these changes were more pronounced in the coconut oil group. In summary, insulin (*P* < 0.004) and C-peptide (*P* < 0.002) decreased in all groups. GIP rose significantly (*P* < 0.040) in all groups. TNF-*α* decreased (*P* < 0.033) with enriched diet, but increased in the lyophilized and coconut oil groups. In contrast, resistin (*P* = 0.085) and adiponectin (*P* = 0.086) did not show significant differences between the groups. Leptin was elevated outside of measurable ranges due to genetic characteristics of db/db mice, as shown in Table 5.

### 3.4. Composition of the Bacterial Community

**Figure 2:**
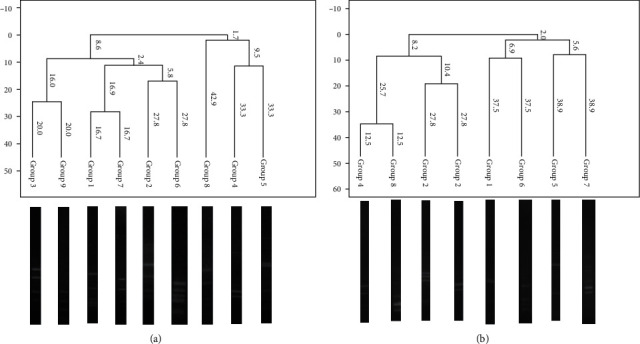
Characterization and comparison of the bacterial communities between groups. Shown are the isolated strains, representatives of each ribotype, like a graphical representation of the distance between the restriction enzyme patterns of the 16S rRNA sequences: (a) Msp-I enzyme (Moraxella sp.) and (b) Rsa-I enzyme (Rhodopseudomonas sphaeroides). Grouping by Sorensen-Dice coefficient and UPGMA.

**Figure 3:**
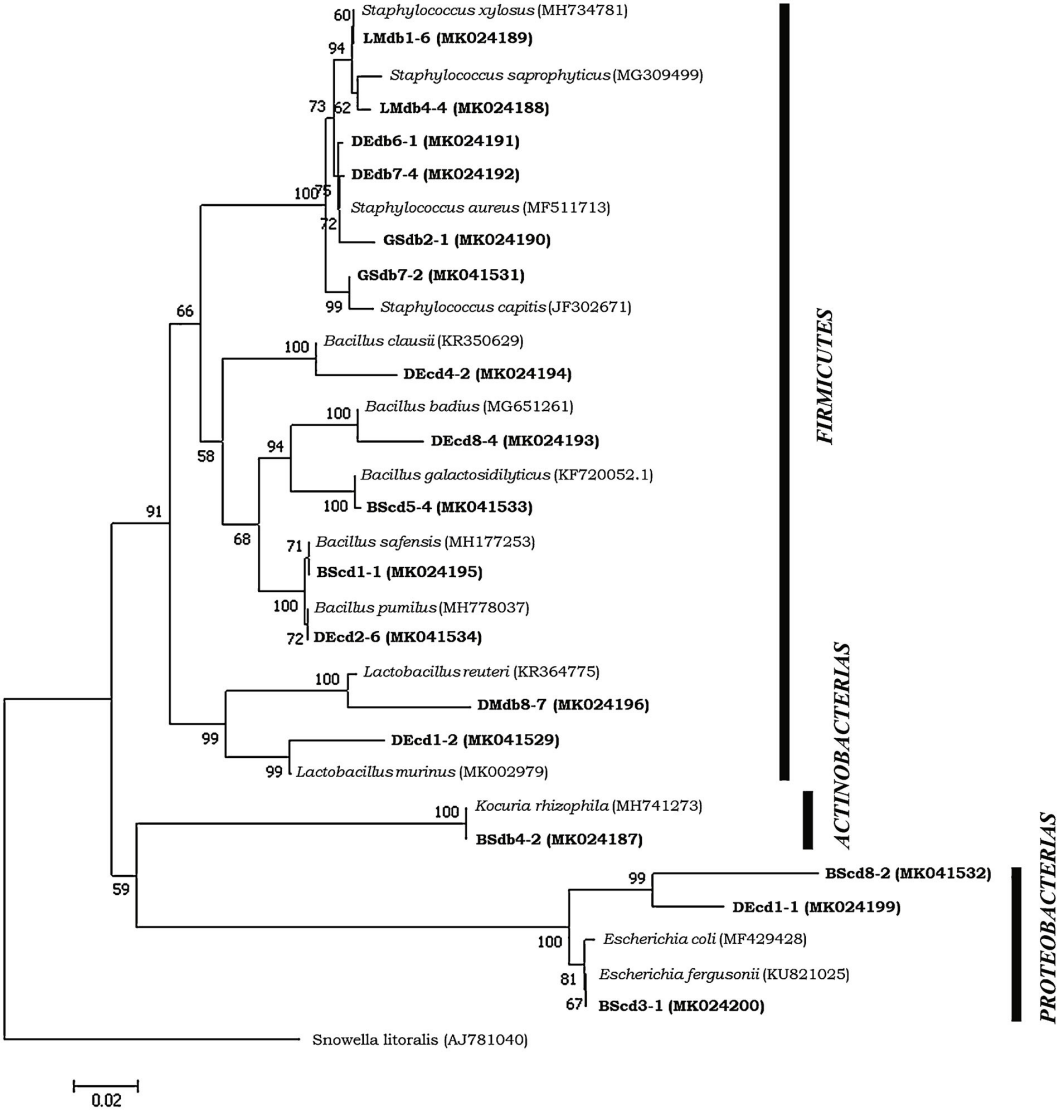
Dendrogram of 9 groups with their respective enzyme restriction profiles. Phylogenetic tree neighbor joining based on sequences of 16S rRNA that shows the phylogenetic relationships of the *Firmicutes* phylum species, *Proteobacteria* and *Actinobacteria*. The Boostrap value of 1000 repetitions is used. The GenBank accession numbers of the 16S rRNA sequences are shown in parentheses. Scale bar corresponds to 20 substitutions for nucleotide positions. The 16S rRNA sequence of the *Cyanobacterium* member was used as an external group (accession number: AJ781040).

The bacterial communities obtained from each group were compared (Figure 2). Representatives of each ribotype were chosen from isolated strains: nine strains from the control group and eight strains from the supplemented groups in BHI culture medium. Overall, of the 16 strains from 32 mice, the most abundant bacteria were *Firmicutes* (75%) followed by *Proteobacteria* (18.75%) and *Actinobacteria* (6.25%) (Figure 3).

### 3.5. Ribotyping

The results of the enzymatic sections revealed that the strains identified by species and genus were grouped into nine different groups: four groups for the supplemented mice and five groups for the control group (Table 2). Figure 3 shows the dendrogram of 9 groups with their respective enzyme restriction profiles. The sequences obtained were compared; five genera and 13 species were identified (Table 2). Table 2 shows the percentages of coverage, coincidence, and similarity obtained through BLAST for each identified species and the diversity of the culture-dependent microbiota of the small intestine.

### 3.6. Phylogenetic Analysis

A phylogenetic tree was constructed based on the 16S rRNA gene sequences of isolated strains and the sequences obtained from GenBank for each genus. Figure 3 shows that five identified genera can be seen. The genus *Bacillus* was the most abundant with five species followed by the genus *Staphylococcus* with four species, *Lactobacillus* and *Escherichia* with two species each one, and finally *Kocuria* with one species (Table 2).

## 4. Discussion

### 4.1. Supplementation with EPA/DHA and Coconut Oil Modified Glycaemia and Food Consumption

The BMI rose in the enriched diet group versus the other groups (lyophilized and coconut oil), but glycaemia and food consumption were lower (Table 3). These results are similar to those reported by Gutiérrez-Pliego et al. [49], who used the same fatty acids derived from microalgae in the diet of db/db mice. However, other studies in C57BL/6J mice and Sprague Dawley rats fed with standard and high *n-3* PUFA diets showed significant differences in the daily amount of food consumed between treatment groups [50, 51]. In contrast, the coconut oil group has increased glycaemia and food consumption with a lower BMI. Coconut oil has been proposed as a functional food to help with weight loss and thereby reduce BMI [21].

### 4.2. The Route of Administration Is More Relevant Than the Type of Fatty Acid Supplied

Total cholesterol was found elevated in all groups of diabetic mice perhaps due to an increase in the synthesis of cholesterol independent of insulin and an increase in circulating VLDL-c with a decrease in LDL-c catabolism via inhibition of the conversion of VLDL-c to LDL-c [52]. The enriched diet increased LDL-c and TG, which is associated with hypertriglyceridemia, low HDL-c levels, and metabolic syndrome [53]. The lyophilized group had no changes in LDL-c, but there was an increase in TG. Hypertriglyceridemia is the most frequent dyslipidemia in diabetics [54]. Supplementation with EPA/DHA derived from microalgae decreased insulin concentration compared to the control group (Table 5). In contrast, the glycaemia behavior was different because it was significantly reduced in the enriched diet group but increased in the lyophilized group. The coconut oil group had the lowest insulin concentration with an increase in blood glucose (Table 3).

One effect of T2DM on the lipid profile is the decreased catabolism of LDL-c by inhibition in the conversion of VLDL-c into LDL-c [55]. This occurs with an enriched diet because it presented high levels of VLDL-c but LDL-c at optimal values. In contrast, the lyophilized group had elevated VLDL-c values with normal LDL-c. However, coconut oil increased LDL-c with lower VLDL-c values than the control group, which is consistent with the study by Sankararaman and Sferra [21]. Another likely explanation may be that the chemical structure of medium-chain fatty acids (MCFAs) of coconut oil allows them to be absorbed in the intestine and sent directly to the liver, making them rapidly available for production of energy; thus, they do not participate in the biosynthesis and transport of cholesterol [56].

Diabetes structurally modifies HDL-c [57], but supplementation with EPA/DHA in a lyophilized form significantly improved HDL-c concentration (Table 4). This shows one of the benefits of consuming supplements with EPA and DHA although other factors do not improve completely because the PAI and CRR increased with a decrease in the CPI. In contrast, the enriched diet group presented low PAI and high CPI with no changes in CRR unlike coconut oil with a low PAI and CPI and elevated CRR.

Total dietary fat intake alters BMI and lipid profile [58], which depends on the type and proportion of fat consumed. For example, SFA consumption increases LDL-c [59], BMI, and insulin resistance [60] causing cognitive decline and Alzheimer's disease [61]. If its consumption is reduced, the LDL-c decreases protecting individuals from cardiovascular events [58]. The EPA and DHA are collectively beneficial in diabetes and obesity because they can inhibit adipocyte hypertrophy and lower the lipid content of adipose tissue [62]. However, coconut oil did have a positive effect with a reduction in VLDL-c and TG (Table 4). While the EPA and DHA reports on its protective and reducing effects on plasma lipids [32, 33] are consistent, our results do not show this. In our study, supplementation modified the composition of total serum lipids and reflected the lipid profile of each diet. The lyophilized group increases HDL-c, VLDL-c, TC, and TG with no changes in LDL-c. These results are consistent with those described by Yoo et al. [62], who administered high-fat diets supplemented with fish oil and microalgae oil and significantly increased HDL cholesterol levels.

The same is seen in Zhukova et al.'s study [63], who observed changes in the lipid profile of Wistar rats supplemented with beef fat (19% of the total diet) and cholesterol (2% of the total diet). These modifications depend on time of exposure to the diet and type of fat administered because this in turn influences the transport and metabolism of lipids in the body.

TC and PAI increased significantly in the lyophilized group, but not with the consumption of enriched diet and coconut oil (Table 4). These results are similar to those of Amaral et al. [64], who showed that the Wistar rats did not modify their total cholesterol levels in the groups treated with diets enriched with fatty fish from the Amazon. In contrast, in a study with Swiss albino mice supplemented with fatty acids derived from the *Caryocar brasiliense* walnut, TC and HDL-c increased with no changes in the atherogenic index and triacylglycerides [65]. The increased of HDL-c demonstrated the protective effect of EPA and DHA of the lyophilized group. This corroborates findings that the lipid profile is modified according to the type and amount of fat consumed. For example, LDL-c decreases significantly in albino rats supplemented with soybean oil [66].

### 4.3. The Enriched Diet and Coconut Oil Altered the Microbiota of the Small Intestine and Hormonal and Inflammatory Profile

The coconut oil decreased the concentration of insulin, adiponectin, and C-peptide with a significant increase in TNF-*α* and GIP (Table 5). There were no modifications in the secretion of resistin. This is consistent with reports of mice fed with high-saturated fat diets that develop inflammation unlike mice fed with fish oil that do not develop metabolic syndrome [67].

**Figure 4:**
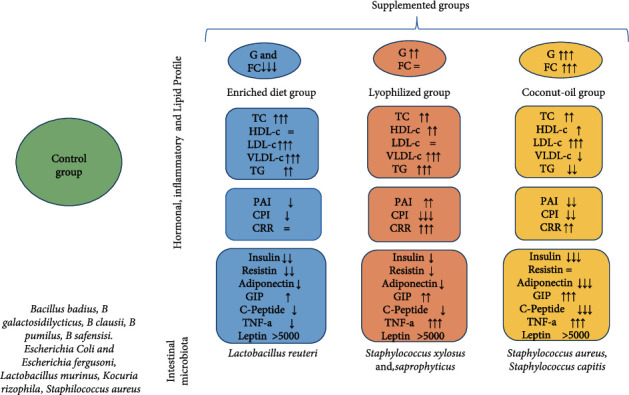
The scheme summarizes and compares the results of metabolic profile and microbiota content of the small intestine in mice supplemented or not for 8 weeks. The enriched diet reduces significantly the hormonal profile, with changes in lipid profile and a significant reduction in TNF-*α* and C-peptide. EPA and DHA administered in lyophilized form induced a higher secretion of TNF-*α* and an increase in atherogenic index and cardio risk ratio, with an increased lipid profile, causing metabolic and inflammatory alterations. Coconut oil moderately increased the lipid profile and reduced C-peptide and hormonal secretion with the exception of GIP, with a significant increase in TNF-*α*. PAI: plasma atherogenic index; CPI: cardioprotective index; CRR: cardiac risk ratio; GIP: gastric inhibitory polypeptide; HDL-c: high-density lipoprotein cholesterol; LDL-c: low-density lipoprotein cholesterol; TC: total cholesterol; TG: triacylglycerides; TNF-*α*: tumor necrosis factor-alpha; VLDL-c: very low-density lipoprotein cholesterol; G: glycaemia; FC: food consumption.

The enriched diet group and the lyophilized group showed similar activity, with a decrease in the secretion of insulin, resistin, adiponectin, and C-peptide and an increase in GIP, TNF-*α* increased only in the lyophilized group. The enriched diet group reduced both C-peptide and TNF-*α*, with a better hormonal-metabolic profile, although the lipid profile was less beneficial (increased TC, LDL-c, VLDL-c, and TG, without changes in HDL-c). A better behavior was observed with the consumption of coconut oil, since it reduced VLDL-c, TG, and PAI, with an increase in HDL-c and a significant decrease in C-peptide, although it has high TNF-*α* (Figure 4). These data provide evidence that dietary supplementation with EPA and DHA derived from microalgae in diabetic mice can support the decrease of the inflammatory state in some parameters such as TNF-*α*, C-peptide, and PAI. This helps to restore metabolic balance and homeostasis and reduces long-term organ damage and therefore complications [68].

### 4.4. The Bacterial Composition Offers Little Diversity

The predominant aerobic bacteria of the small intestine were identified. *Bacillus* bacteria were detected in the control group. Some species belonging to this genus are known as pathogens or opportunists [69] including *Bacillus clausii* (a probiotic capable of modulating the immune response) [70]; *Bacillus pumilus* and *Bacillus safensis* have probiotic effects [71]. *Bacillus galactosidilyticus* and *Bacillus badius* have been found in feces of Balb/c mice fed on organic components; the main activity of *Bacillus* has been described like a probiotic [72]; therefore, EPA and DHA may be favoring their proliferation and activity in the body. However, not all probiotic bacteria exert the same action within the host, and not all use the same strategies to beat competing microorganisms in the gastrointestinal tract [72]. Most *Bacilli* are found in the small intestine to promote the processes of absorption, digestion, and use of nutrients; therefore, it is safe to agree that the diet used for control mice is ideal for maintenance of the microbiota in the small intestine.

In the small intestine of the control group, *Bacillus badius*, *galactosidilyticus*, *clausii*, *pumilus*, *safensis*, *Escherichia coli*, *Escherichia fergusonii*, *Lactobacillus murinus*, *Kocuria rhizophila*, and *Staphylococcus aureus* were found. The proportion and diversity of bacteria in the supplemented groups were markedly reduced. In the enriched diet group, only *Lactobacillus reuteri* was found. Different diversities were observed in the lyophilized group with *Staphylococcus xylosus* and *Staphylococcus saprophyticus* as well as the group supplemented with coconut oil (*Staphylococcus aureus* and *Staphylococcus capitis*) as shown in Figure 4. The most abundant group of bacteria found in this study was *Firmicutes* (75%).

Current evidence from genomic studies of the gut microbiota in mice shows that the most abundant groups in *Phylum* are *Firmicutes* (60-80%) and *Bacteroidetes* (20-30%) [73]. This trend is modified in T2DM: the percentage of *Firmicutes* increases and *Bacteroidetes* decreases [3]. Most studies describe the microbiota of the large intestine obtained from feces. Perhaps for this reason, we did not find *Bacteroidetes* because the microbiota was analyzed from solids obtained from the small intestine. In reports of mice supplemented with high-fat diets, primarily safflower oil, the abundance of *Bacteroidetes* decreased and colonization of *Firmicutes*, *Actinobacteria*, and *Proteobacteria* increased similar to our findings [74].

The strains reported in the study by Muñoz-Garach et al. [3], in patients with T2DM, which are *Lactobacillus reuteri*, *Streptococcus mutans*, *and Escherichia coli*, coincide with the results of this study. The most abundant genus in diabetic mice was *Staphylococcus*; these can be harmless commensal pathogens [75]. Another species found was *Staphylococcus aureus*, which is an opportunistic pathogen in mice [76]. The duodenum has a low bacterial count and is mainly colonized by aerobic bacteria whereas anaerobic organisms appear in the terminal ileum for their continuity with the colon [77]. These findings can explain the presence of *Staphylococcus aureus* in diabetic mice in this study. On the other hand, *Staphylococcus xylosus* is a common commensal bacterium that is found in the mucous membranes of mammals. It can proliferate in an immunosuppressed host as in T2DM [78]. Another pathogen found in diabetic mice belongs to the *Actinobacteria* (phylum of *Micrococcaceae* family *Kocuria rhizophila*) isolated from a wide variety of natural sources such as soil, fresh water, and fish intestines [79]. Its function is to produce gastric enzymes, and it is sensitive to antibiotics [80]. Another genus of the small intestine is *Lactobacillus reuteri* that is also reported in the small intestine of FvB mice [79]. This is a probiotic bacterium that improves the absorption of micronutrients, modulates the host's immune responses, promotes the integrity of the intestinal mucosa, and reduces bacterial translocation [81]. *Lactobacillus reuteri* has also been used in the remodeling of the microbiota of SF mice to reduce inflammation of multiple organs [82]. Therefore, its presence in the enriched diet group in the present study can be considered positive because it reflects an improvement in the inflammatory response of the T2DM model applied in this study.

Another genus found through the 16S rRNA analysis was *Enterococcus hirae*, and this is a commensal and nonpathogenic microorganism present in the gastrointestinal tract. This species has potential probiotic characteristics and is resistant to acidic conditions, bile salts, and gastric juice [80]. Finally, the genus *Escherichia* was seen mainly *Escherichia coli* and *Escherichia fergusonii*. The first colonizes the intestine of humans and animals and may be pathogenic and cause disease or may help keep the digestive system healthy [83]. The latter is associated with a wide variety of intestinal infections in both humans and animals [84].

The modification of the bacterial community of the small intestine, between db/db groups of mice, can be attributed to two factors: (1) the type of fatty acid administered (EPA/DHA) and coconut oil and (2) the way it was administered (either added to the food, diluted in water, or in the oil form). This reflects a decrease in bacterial diversity, composition, and abundance, perhaps reflecting the hormonal and metabolic disturbances *per se* derived from T2DM. This agrees with reported data where it is mentioned that obesity, insulin resistance, fatty liver, and low degree of inflammation (increased C-peptide and leptin with decreased concentration of adiponectin) cause a low diversity of the intestinal microbiota [80]. This confirms that diet plays a preponderant role in the composition and modification of the microbiota [80]; thus, the consumption of EPA/DHA and coconut oil in db/db mice leads to modifications in the hormonal and inflammatory profiles with changes in the intestinal microbiota. However, modifications are different in each case although they all decrease bacterial diversity. Coconut oil actually improves the inflammatory and metabolic profile but not the lipid profile. This can be explained in part because coconut oil has better implications for the host's energy balance compared to lipids rich in long-chain fatty acids [83]. Even a high-soy diet has been shown to be more detrimental to metabolic health than high-coconut oil diet [85, 86].

The lyophilized group improves the lipid profile but is not beneficial to the hormonal and inflammatory profile. A protective effect of fish oil against intestinal microbial dysbiosis, endotoxemia, and inflammation has been suggested in several studies [35, 87, 88]. The endotoxemia and dysbiosis also have been observed with the administration of butter-based fats that altered the composition of the intestinal microbiota (*Enterobacteriaceae* increased and *Bifidobacteria* decreased) with increased permeability and high levels of endotoxins in the intestine, in comparison to those fed *n-6* and *n-3* polyunsaturated fatty acids [89]. In fact, the only one that does not show an improvement in any of the parameters is the administration of the enriched diet perhaps because the treatment was diluted in food; this could reduce its absorption capacity or use by the body. Diets enriched with soybean oil have a significant effect on the caecal microbiota of newly weaned mice, which represents an early consequence of increased dietary fat [67]. On the other hand, C57BL/6N mice that received diets enriched with coconut oil had modified intestinal microbiota, microbial metabolic pathways, and lipid metabolism. These features modulated specific bacterial populations [67]. The use of coconut oil continues to be controversial, and some studies report that the addition of this oil to the diet improves the antioxidant state and immunity [90]. Despite this, there is no consensus regarding the beneficial use of coconut oil.

## 5. Conclusions

Supplementation with EPA and DHA derived from microalgae added to the diet reduced TNF-*α* and C-peptide, which contributes to improving the systemic inflammatory status, but depressed the diversity of the small intestine microbiota in diabetic mice. In contrast, coconut oil only decreased the C-peptide, raising TNF- *α*, with an unfavorable hormonal and lipid profile. The presence of *Firmicutes*, *Actinobacteria*, and *Proteobacteria* was found. Indeed, the *Firmicutes* were the most abundant, while the *Proteobacteria* showed the least abundance. The consumption of EPA/DHA derived from microalgae should be included in the diet for optimal use. The effect of coconut oil consumption, despite raising the HDL-c fraction and reducing TG, has harmful effects on other parameters such as TNF-*α* elevation. This can be translated into an adaptation of the organism due to the changes produced by the different dietary patterns. The differences found between the groups could be due to the different ways in which fatty acids were administered. In spite of it not being possible to transfer the results of the animal models to the clinical setting, it is possible to mention the relevance and future perspective. In this sense, the administration of EPA and DHA in this model of diabetic mice allows us to glimpse its effect at the systemic level, but is not clear whether it has an impact on the intestinal microbiota.

## Figures and Tables

**Table 1:** Nutritional content of the standard diet, compared to the enriched diet.

Macronutrients		SD	ED
		%	%
Fatty acids	Linoleic acid	1.22	1.22
	Linolenic acid	0.10	0.10
	Arachidonic acid	<0.01	<0.01
	*EPA and DHA*	0.19	**2.19** ^∗^
	Total saturated fatty acids	1.56	1.37
	Total monounsaturated fatty acids	1.6	1.6
	Total fatty acids	4.68	6.49
	Other fats^∗^	6.02	6.21
Total lipids		**10.7**	**12.7** ^∗^
Carbohydrates		48.7	47.21
Proteins		23.98	23.48
	Total	83.38	83.39
Micronutrients and fiber		16.62	16.61
	Total	100	100

Standard diet (SD): Rodent Laboratory Chow® 5001 from Purina. ^∗^Enriched diet (ED): with 2% more EPA and DHA. ^∗^Other fats include cholesterol, 200 ppm.

**Table 2:** Ribotypological grouping and BLASTn identification of 16S rRNA sequences distributed according to the number of bands.

Sample number	Strain code^∗^	Characterization RFLP-PCR^∗∗^		Identification by BLASTn	Access no. GenBank	Coverture (%)	Similarity (%)
		Rsa-I	Msp-I				
*Control group*							
1	DEcd8-4	4	5	*Bacillus badius*	MK024193	99	94
2	DEcd4-2	5	4	*Bacillus clausii*	MK024194	97	90
2	BScd5-4	5	4	*Bacillus galactosidilyticus*	MK041533	100	99
3	DEcd2-6	4	6	*Bacillus pumilus*	MK041534	100	99
4	BScd1-1	4	3	*Bacillus safensis*	MK024195	99	98
1	BScd8-2	4	5	*Escherichia coli*	MK041532	100	91
1	DEcd1-1	4	5	*Escherichia coli*	MK024199	91	90
1	BScd3-1	4	5	*Escherichia fergusonii*	MK024200	99	95
5	DEcd1-2	4	3	*Lactobacillus murinus*	MK041529	93	97
6	BSdb4-2	4	5	*Kocuria rhizophila*	MK024187	100	98
7	DEdb7-4	5	4	*Staphylococcus aureus*	MK024192	98	95
7	DEdb6-1	5	4	*Staphylococcus aureus*	MK024191	100	99
*Enriched diet group*							
9	DMdb8-7	—	4	*Lactobacillus reuteri*	MK024196	100	92
*Lyophilized group*							
8	LMdb4-4	4	4	*Staphylococcus saprophyticus*	MK024188	99	97
*Coconut oil group*							
7	GSdb2-1	5	4	*Staphylococcus aureus*	MK024190	99	97
7	GSdb7-2	5	4	*Staphylococcus capitis*	MK041531	100	98

Identification codes were assigned to the samples. ^∗^Strain samples ordered for RFLP groups. ^∗∗^Number of bands in agreement to enzymatic cuts. The first two initials correspond to the treatment control group (BScd, DEcd, and DEdb), lyophilized group (LMdb), enriched diet group (DMdb), and coconut oil group (GSdb) and finally the mouse number. The percentages of coverage and similarity are greater than 90%; the lower the *e* value, the alignment is more significant.

**Table 3:** BMI, glycaemia, and food consumption in db/db mice supplemented with EPA/DHA derived from microalgae and coconut oil.

	CLG	EDG	LG	COG	
	Mean ± SD	Mean ± SD	Mean ± SD	Mean ± SD	*P* value
BMI (g/cm^2^)	62 ± 2.5	63.8 ± 1.3	62.8 ± 1.3	60.1 ± 2.5	0.756
Glycaemia (mg/dL)	539.5 ± 7.3	518 ± 5.4+	544.6 ± 41	584 ± 19+	0.001^∗^
Food consumption (g)	32.7 ± 0.33	28.8 ± 2.0+	31.9 ± 7.4	36.1 ± 1.8+	0.012^∗^

The data represent the mean ± SD of BMI, glycaemia, and food consumption of mice supplemented with EPA/DHA derived from microalgae and coconut oil, for 8 weeks. One-way ANOVA^∗^ was performed, with Tukey's+post *hoc* test, to compare the groups; a *P* < 0.05 was considered significant. BMI: body mass index; CLG: control group; EDG: enriched diet group; LG: lyophilized group; COG: coconut oil group.

**Table 4:** Lipid profile of supplemented mice for 8 weeks.

	CLG	EDG	LG	COG	
	Median (mg/dL)	Median (mg/dL)	Median (mg/dL)	Median (mg/dL)	*P* value
TC	137	250	233	228	0.003^∗∗^
HDL-c	53.1	54.6	95.4	58.8	0.015^∗∗^
LDL-c	50.7	150	59.4	128.1	0.004^∗∗^
VLDL-c	35.6	46.6	60	31.8	0.003^∗∗^
TG	178	233	300	159	0.003^∗∗^
PAI	0.525	0.497	0.630	0.437	0.007^∗∗^
CPI	1.04	1.6	0.364	0.459	0.001^∗∗^
CRR	2.5	2.44	4.57	3.87	0.001^∗∗^

The data represent the median of the lipid profile values of the supplemented and control groups, during 8 weeks. The nonparametric test of Kruskal-Wallis^∗∗^ was carried out to make the comparison between groups. The *P* value < 0.05 was considered significant. TC: total cholesterol; HDL-c: high-density lipoprotein cholesterol; LDL-c: low-density lipoprotein cholesterol; VLDL-c: very low-density lipoprotein cholesterol; TG: triacylglycerols; PAI: plasma atherogenic index; CPI: cardioprotective index; CRR: cardiac risk ratio; CLG: control group; EDG: enriched diet group; LG: lyophilized group; COG: coconut oil group.

**Table 5:** Hormone and inflammatory profile of supplemented and control mice for 8 weeks.

	CLG	EDG	LG	COG	
	Median (pg/dL)	Median (pg/dL)	Median (pg/dL)	Median (pg/dL)	*P* value
Insulin	18.614	14,076	15,183	12,831	0.004^∗∗^
Resistin	6,512	5,111	5,355	6,502	0.085
Adiponectin	1,476	1,397	1,316	1,225	0.086
GIP	226	236	263	499	0.040^∗∗^
C-Peptide	5,762	5,068	5,475	4,631	0.002^∗∗^
TNF-*α*	30.6	27.1	42.7	62	0.033^∗∗^
Leptin	>5,000	>5,000	>5,000	>5,000	0.893

The data represent the median of hormone and inflammatory profile of the supplemented groups, during 8 weeks. Values represent the median of data expressed in pg/dL of hormonal and inflammatory profile. The nonparametric test of Kruskal-Wallis^∗∗^ was carried out to compare the groups. The data were considered significant with a *P* value < 0.05. GIP: glucose-dependent insulinotropic polypeptide; TNF-*α*: tumor necrosis factor alpha; CLG: control group; EDG: enriched diet group; LG: lyophilized group; COG: coconut oil group.

## Data Availability

The standards have been revised, the data is openly available on GenBank, and the rest of the data can be requested from the corresponding author.
